# EGFR Signaling Causes Morphine Tolerance and Mechanical Sensitization in Rats

**DOI:** 10.1523/ENEURO.0460-18.2020

**Published:** 2020-04-06

**Authors:** Stephanie Puig, Courtney L. Donica, Howard B. Gutstein

**Affiliations:** 1 Department of Anesthesiology, University of Pittsburgh School of Medicine, Pittsburgh, PA 15213; 2 University of Houston, Houston, TX 77030; 3 Anesthesiology Institute, Allegheny Health Network, Pittsburgh, PA 15224

**Keywords:** growth factors, morphine, neuropathic, opioids, pain, tolerance

## Abstract

The safety and efficacy of opioids are compromised as analgesic tolerance develops. Opioids are also ineffective against neuropathic pain. Recent reports have suggested that inhibitors of the epidermal growth factor receptor (EGFR), a receptor tyrosine kinase (RTK), may have analgesic effects in cancer patients suffering from neuropathic pain. It has been shown that the platelet-derived growth factor receptor-β (PDGFR-β), an RTK that has been shown to interact with the EGFR, mediates opioid tolerance but does not induce analgesia. Therefore, we sought to determine whether EGFR signaling was involved in opioid tolerance and whether EGFR and PDGFR signaling could induce pain in rats. We found that gefitinib, an EGFR antagonist, eliminated morphine tolerance. In addition, repeated EGF administration rendered animals unresponsive to subsequent analgesic doses of morphine, a phenomenon we call “pre-tolerance.” Using a nerve injury model, we found that gefitinib alone was not analgesic. Rather, it reversed insensitivity to morphine analgesia (pre-tolerance) caused by the release of EGF by injured nerves. We also showed that repeated, but not acute EGF or PDGF-BB administration induced mechanical hypersensitivity in rats. EGFR and PDGFR-β signaling interacted to produce this sensitization. EGFR was widely expressed in primary sensory afferent cell bodies, demonstrating a neuroanatomical substrate for our findings. Taken together, our results suggest a direct mechanistic link between opioid tolerance and mechanical sensitization. EGFR antagonism could eventually play an important clinical role in the treatment of opioid tolerance and neuropathic pain that is refractory to opioid treatment.

## Abstract (precis)

**Figure:**
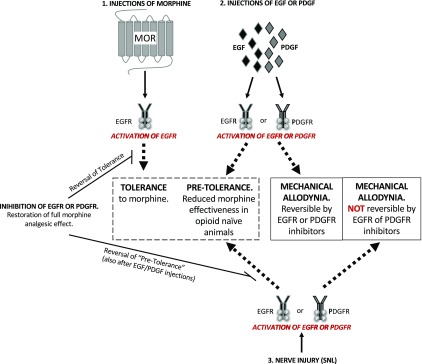

## Significance Statement

Opioid tolerance and associated reduced effectiveness of opioids against neuropathic pain are two major clinical problems that are prime contributors to the opioid epidemic. However, the mechanisms underlying these phenomena are not clearly understood. Here, we show that epidermal growth factor receptor (EGFR) antagonism not only blocks morphine tolerance but also restores the effectiveness of opioids against neuropathic pain. Chronic EGF or platelet-derived growth factor (PDGF) administration induces mechanical sensitization, a prominent component of neuropathic pain, and renders animals “pre-tolerant” to subsequent analgesic doses of morphine. Taken together, these results suggest a direct mechanistic link between opioid tolerance and neuropathic pain. EGFR antagonism could eventually play an important role in the treatment of opioid tolerance and severe neuropathic pain that requires ever increasing doses of opioids.

## Introduction

For centuries, opioid drugs such as morphine have been the first-line treatment for severe chronic pain. However, over time tolerance to opioid analgesia develops. Because there are few alternatives to opioids for the treatment of chronic severe pain, marked increases in opioid dose may be required to compensate for inadequate analgesia as tolerance develops. However, tolerance to the unpleasant or potentially life-threatening side effects of opioids such as respiratory depression, constipation, urinary retention, and delirium, does not occur as rapidly as analgesic tolerance (Collett, 1998; Gutstein and Akil, 2006). Therefore, patients face increased risk as well as suffering when opioids lose effectiveness. Despite extensive research, safer and more effective options for the treatment of severe chronic pain have not been found (Kissin, 2010). In addition, opioids may not be effective against pain due to nerve injury (neuropathic pain; Woolf and Mannion, 1999; Donica et al., 2014). The reasons for this resistance to opioid analgesia are unclear. However, pain and opioid tolerance have long been hypothesized to share common underlying mechanisms (Mayer et al., 1999; Joseph et al., 2010), suggesting that there could be a potential link between the two phenomena.

The epidermal growth factor receptor (EGFR) is a receptor tyrosine kinase (RTK) expressed in the nervous system (Huerta et al., 1996; Pearson and Carroll, 2004; Wong and Guillaud, 2004) that can be activated *in vitro* by the μ-opioid receptor (MOR; Belcheva et al., 2001, 2003). Recent case reports have suggested that EGFR inhibitors markedly reduced neuropathic pain in cancer patients (Kersten and Cameron, 2012; Kersten et al., 2015). It has also been suggested that EGFR may be involved in pain and analgesia signaling in rats (Martin et al., 2017). Previously it has been shown that the platelet-derived growth factor receptor (PDGFR), an RTK that interacts with the EGFR (Habib et al., 1998; Saito et al., 2001), mediates opioid tolerance (Wang et al., 2012) and can induce allodynia (Masuda et al., 2009). Therefore, we wondered whether the EGFR could also be involved in the mechanisms underlying opioid tolerance and if PDGFR and EGFR could interact to mediate pain and modulate analgesia.

In this study, we made several important discoveries. First, we showed that the EGFR is specifically expressed in dorsal root ganglia (DRG) neurons and in the dorsal horn of the spinal cord, areas of key importance in the modulation of pain and analgesia. We then demonstrated that gefitinib, a clinically approved EGFR inhibitor, prevented and reversed morphine tolerance in rats. In addition, we observed that after repeated EGF administration, animals became unresponsive to subsequent analgesic doses of morphine, a phenomenon we called “pre-tolerance.” These findings implied that EGFR signaling was both necessary to observe and sufficient to induce morphine tolerance. We also used the sciatic nerve ligation (SNL) model to explore the role of EGFR signaling in chronic neuropathic pain. Gefitinib was not analgesic in the nerve injury model. Rather, it reversed EGF-induced insensitivity to morphine analgesia (pre-tolerance). We also found that repeated administration of either EGF or PDGF-BB induced mechanical allodynia. Neither EGF nor PDGF-BB induced thermal sensitization, suggesting that these two growth factors induce modality specific hypersensitivity. Interestingly, EGFR and PDGFR signaling interacted in the generation of mechanical allodynia caused by repeated EGF or PDGF-BB.

In sum, our findings show that EGFR inhibition not only blocks morphine tolerance but also restores the effectiveness of opioids against neuropathic pain, suggesting a direct mechanistic link between opioid tolerance and neuropathic pain. EGFR antagonists could eventually play an important role in the treatment of opioid tolerance and severe neuropathic pain that is refractory to opioid treatment.

## Materials and Methods

### Animals

Male Sprague Dawley rats (175–200 g, Harlan) were housed in groups of three and were maintained on a 12/12 h light/dark cycle with *ad libitum* access to food and water. Rats habituated to the colony room for one week prior to experimental manipulations. All protocols were approved by the MD Anderson and University of Pittsburgh Animal Care and Use Committee.

### Drug administration

Drugs were dissolved in a solution of 10% β-cyclodextrin sulfobutyl ether (Captisol, CyDex) solution and 0.9% saline. Morphine sulfate was obtained from the MD Anderson and University of Pittsburgh pharmacy and Sigma, gefitinib from LC Laboratories, recombinant rat EGF peptide, recombinant rat PDGF-BB peptide and recombinant human EGFR-Fc scavenger from R&D Systems. EGF and PDGF-BB peptides were reconstituted at 100 μg/ml in sterile 10 mm acetic acid or in sterile 4 mM HCl respectively and stored at −8°C until used. The final HCl concentration in control, EGF, and PDGF-BB containing solutions was 0.5 mm. The EGFR-Fc scavenger was re-constituted in PBS with 0.1% bovine serum albumin (BSA) at 100 μg/ml and stored at −80°C until use. Drugs were administered daily via subcutaneous injection (1 mg/ml, w/v) or lumbar puncture (20 μl per injection) as previously described (Xu et al., 2006).

### Spinal nerve ligation

Left L5 spinal nerve ligations were performed as described previously (Chung et al., 2004). Briefly, animals were anesthetized with 2.5% isoflurane. After locating the L6 vertebra, a small incision in the skin was made on the left side of the vertebra and muscle was moved aside to expose the L5 root of the sciatic nerve. A tight ligation was performed around the nerve before closing the wound. Animals were allowed a week for recovery before the beginning of the experiments. Sham animals underwent the same type of surgery, exposing the L5 nerve root, but the wound was closed without ligating the nerve.

### Nociceptive testing

For mechanical sensitivity assessment, animals were placed in Plexiglas cages on a mesh surface and habituated for 30 min/d for 3 d prior to testing. Mechanical sensitivity was assessed by Von Frey filaments (Stoelting) using the up-down method of Dixon and median 50% threshold determined as described (Dixon, 1980; Chaplan et al., 1994). Thermal sensitivity and morphine analgesia was assessed using the radiant heat tail flick latency (TFL) or paw withdrawal latency (PWL) tests. Animals were placed in Plexiglas cages on a modified Hargreaves device (UCSD) with a constant surface temperature of 30°C. Rats were habituated to the device for 30 min/d for 3 d before testing. A hot lamp was focused on the tail and reflex withdrawal time was determined by a photocell. 10 s was used as a cutoff to avoid tissue damage to the tail. TFL were measured 40 min after intrathecal or subcutaneous injection. The same device was used for PWL, with a 20-s cutoff time.

### Immunohistochemistry

Spinal cords were dissected from naive rats. The lumbar portion of fresh spinal cords were cut into 3 mm thick cross-sectional pieces and postfixed in 2% PFA in PBS for 48 h. DRGs were collected from naive rats that were anesthetized and perfused with normal saline followed by 4% PFA in PBS. The spinal cord and lumbar DRG were dissected and postfixed overnight in 4% PFA. All tissue samples were then transferred to 20% and 30% sucrose diluted in 0.1 M PBS for cryoprotection. Tissue equilibrated in embedding matrix (OCT, TissueTek) was snap frozen in isopentane (−55°C) and stored at −80°C. Frozen tissue samples were sectioned using a cryostat. Spinal cord sections (25 μm) were processed floating in PBS at room temperature (RT). DRG sections (10 μm) were mounted on super frost + slides (Fisher), dried overnight at RT and stored at −8°C until used. Tissue was rinsed 2 × 5 min then blocked in 5% normal goat serum (NGS) containing 0.2% Triton X-100 (TX100) with PBS for 1 h at RT. Primary antibody incubation was done in a 1% NGS, 0.2% TX100, PBS buffer, overnight at 4°C. Primary antibodies used: anti-EGFR, rabbit polyclonal, 1:500 (Santa Cruz Biotechnology); anti-CGRP, mouse monoclonal, 1:500 (Abcam); anti-NF200, mouse monoclonal, 1:2000 (Sigma-Aldrich); anti-GFAP, mouse monoclonal, 1:750 (Millipore); anti- NeuN, mouse monoclonal, 1:1000 (Millipore); anti-CD11 OX42, mouse monoclonal, 1:750 (Millipore); and Isolectin-B4 Alexa Fluor 568 conjugated, 1:1000 (Life Technologies). After rinsing 3 × 5 min with PBS tissue was incubated with Alexa Fluor 488 goat anti-rabbit IgG and Alexa Fluor 568 goat anti-mouse (1:2000, Life technologies) secondary antibodies diluted in 2% NGS in PBS in the dark at RT for 1 h. Tissue was then rinsed 3 × 5 min. with PBS, incubated with DAPI (50 ng/ml in PBS, Cell Signaling Technologies) 5 min at RT and rinsed 3 × 5 min. Finally, sections were mounted, air dried, coverslipped in Prolong Gold antifade mounting media (Invitrogen Molecular Probes), and stored at 4°C. Imaging was performed using a confocal microscope, Nikon A1.

### Data analyses

The experimenter was blinded to treatment group throughout all the experiments. Behavioral data were analyzed using GraphPad Prism 7.0 (RRID:SCR_002798). The Shapiro–Wilk normality test was performed. Data were then analyzed using a two-way ANOVA followed by Dunnett’s multiple comparison *post hoc* analysis and considered statistically significant if *p* < 0.05. All statistical tests used, and resultant confidence intervals are presented in Table 1.

**Table 1.** Statistical analyses used within the manuscript

	Data structure	Type of test	Comparison	95% confidence interval
Figure 2*A*	Non-normally distributed	Two-way ANOVA		
		Dunett's multiple comparison test	Morphine vs vehicle	0.864 to 1.719
			Morphine vs gefitinib	0.7783 to 2.011
			Morphine vs morphine + gefitinib	–2.349 to –1.117
Figure 2*B*	Non-normally distributed	Two-way ANOVA		
		Dunett's multiple comparison test	Morphine vs vehicle	0.8614 to 1.635
			Morphine vs gefitinib	0.7111 to 1.484
			Morphine vs morphine + gefitinib	–2.034 to –1.223
Figure 2*C*	Non-normally distributed	Two-way ANOVA		
		Dunett's multiple comparison test	Morphine vs vehicle	0.1181 to 1.828
			Morphine vs EGF	0.8573 to 2.567
Figure 3*A*	Non-normally distributed	Two-way ANOVA		
		Dunett's multiple comparison test	Morphine vs sham	–11.35 to –9.522
			Morphine vs gefitinib	–0.9836 to 0.9314
			Morphine vs morphine + gefitinib	–8.873 to –6.958
Figure 3*B*	Normally distributed	Two-way ANOVA		
		Dunett's multiple comparison test	Morphine vs vehicle	–1.497 to 1.603
			Morphine vs EGF-FC	–1.904 to 1.196
			Morphine vs morphine + EGF-FC	–10.45 to –7.348
Figure 4*A*	Normally distributed	Two-way ANOVA		
		Dunett's multiple comparison test	Vehicle vs imatinib	–1.891 to 2.498
			Vehicle vs EGF	6.313 to 10.07
			Vehicle vs EGF+ imatinib	–6913 to 3.697
Figure 4*B*	Normally distributed	Two-way ANOVA		
		Dunett's multiple comparison test	Vehicle vs imatinib	–1.515 to 1.977
			Vehicle vs EGF	–1.367 to 2.126
			Vehicle vs EGF+ imatinib	–1.732 to 1.761
Figure 4*C*	Normally distributed	Two-way ANOVA		
		Dunett's multiple comparison test	Vehicle vs gefitinib	–1.408 to 2.104
			Vehicle vs PDGF	4.401 to 7.913
			Vehicle vs PDGF+ gefitinib	–0.1661 to 3.346
Figure 4*D*	Normally distributed	Two-way ANOVA		
		Dunett's multiple comparison test	Vehicle vs gefitinib	–0.921 to 1.743
			Vehicle vs PDGF	–0.9921 to 1.536
			Vehicle vs PDGF+ gefitinib	–0.606 to 2.058

## Results

**Figure 1.**
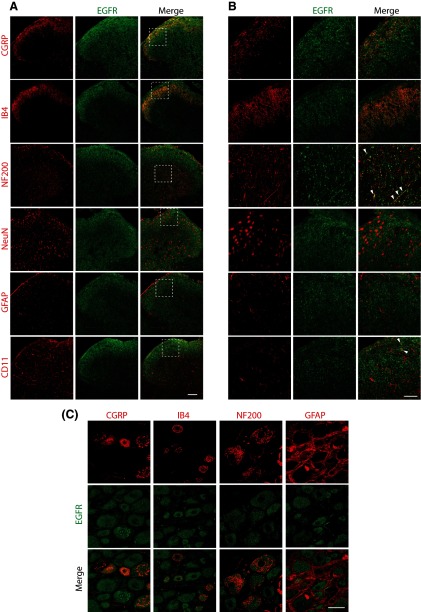
EGFR expression in the spinal cord and DRG. ***A***, EGFR expression (green) was concentrated in the SG of the spinal cord and colocalized with CGRP, IB4, OX42, and NF200. 20× objective. Scale bar = 100 μm. ***B***, Higher magnification images of the boxed regions in panel ***A*** using a 60× objective lens demonstrated that EGFR co-localized with unmyelinated peptidergic (CGRP) and unmyelinated non-peptidergic (IB4) primary sensory afferent terminals in the SG. EGFR also co-localized with myelinated (NF200, arrowheads) primary sensory afferent terminals in in deeper layers of the dorsal horn of the spinal cord. The EGFR did not co-localize with neuronal cell bodies (NeuN) or astrocytes (GFAP) in the SG. Sparse co-localization within microglial cell bodies (OX42, arrowheads) was also observed in the SG. 60× objective. Scale bar = 50 μm. ***C***, EGFR co-localized with unmyelinated non-peptidergic (IB4), unmyelinated peptidergic (CGRP) and myelinated (NF200) sensory primary afferent neuronal cell bodies in the DRG. The EGFR did not co-localize with GFAP-expressing satellite cells; 40× objective. Scale bar = 20 μm. Nikon A1 confocal microscope.

We found that the EGFR was expressed in both the spinal cord and DRG. Spinal cord expression was widespread, but highly concentrated in the substantia gelatinosa (SG; Fig. 1*A*). Co-immunostaining of EGFR with markers for different types of primary afferent sensory neurons in the SG revealed that the EGFR co-localized with unmyelinated peptidergic (CGRP+) fibers as well as with unmyelinated non-peptidergic (IB4+) and with some larger-diameter myelinated (NF200+) primary afferent fibers (Fig. 1*B*). Colocalization studies with markers for SG neurons (NeuN), astrocytes (GFAP) and microglia (OX42) revealed that the EGFR did not colocalize within neurons or astrocytes in the SG, but did show sparse colocalization within microglia (Fig. 1*B*). In the DRG, the EGFR did co-localize with cell bodies expressing CGRP, IB4, and NF200. However, it did not co-localize with GFAP, a marker for satellite cells (Fig. 1*C*).

**Figure 2.**
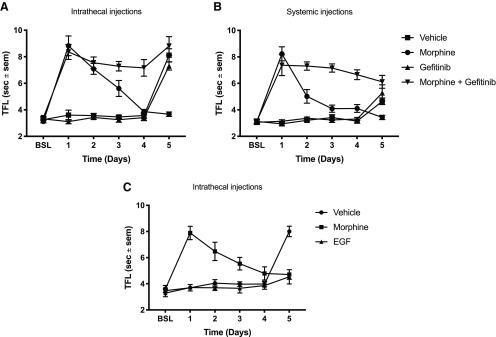
EGFR activation is both necessary and sufficient to cause morphine tolerance. ***A***, Rats received daily intrathecal injections of either 455 ng morphine, 10 μg gefitinib, MS + gefitinib or vehicle for 4 d. On day 5, all animals received morphine alone; *N* = 6 rats per group, two-way ANOVA, interaction: *F*_(15,100)_ = 20.92, *p* < 0.0001, days: *F*_(5,100)_ = 47.51, *p* < 0.0001, treatment: *F*_(3,20)_ = 68.17, *p* < 0.0001. ***B***, Rats received daily subcutaneous injections of either 3.5 mg/kg morphine, 5 mg/kg gefitinib, MS + gefitinib or vehicle for 5 d. On day 6, all animals received MS alone; *N* = 5–6 rats per group, two-way ANOVA, interaction: *F*_(15,95)_ = 14.75, *p* < 0.0001; days: *F*_(5,95)_ = 21.87, *p* < 0.0001; treatment: *F*_(3,19)_ = 166.4, *p* < 0.0001. ***C***, Animals received daily intrathecal injections of either 455 ng MS, 63 ng EGF, or vehicle for 4 d. On day 5, all animals received MS alone; *N* = 6 rats per group, two-way ANOVA, interaction: *F*_(10,75)_ = 19.45, *p* < 0.0001; days: *F*_(5,75)_ = 20.43, *p* < 0.0001; treatment: *F*_(2,15)_ = 12.01, *p* < 0.0001.

To determine whether EGFR signaling could modulate tolerance, rats were injected daily with morphine in the presence or absence of the EGFR inhibitor gefitinib. As expected, repeated morphine injections induced analgesic tolerance. However, co-administration of morphine and gefitinib completely eliminated morphine tolerance when administered intrathecally (vehicle: Captisol 10%, morphine: 455 ng, gefitinib: 10 μg, *N* = 6 rats per group, two-way ANOVA, interaction: *F*_(15,100)_ = 20.92, *p* < 0.0001; days: *F*_(5,100)_ = 47.51, *p* < 0.0001; treatment: *F*_(3,20)_ = 68.17, *p* < 0.0001; Fig. 2*A*) or systemically (vehicle: Captisol 10%, morphine: 3.5 mg/kg, gefitinib: 5 mg/kg, *N* = 5–6 rats per group, two-way ANOVA, interaction: *F*_(15,95)_ = 14.75, *p* < 0.0001; days: *F*_(5,95)_ = 21.87, *p* < 0.0001; treatment: *F*_(3,19)_ = 166.4, *p* < 0.0001; Fig. 2*B*).The fact that co-administration of gefitinib did not alter the analgesic effect of morphine on day 1 of administration suggests that the effect of gefitinib selectively targeted the tolerance-inducing properties of morphine (Fig. 2*A*,*B*). Repeated administration of gefitinib alone did not cause analgesia. On the last day of the experiments, all animals were injected with morphine alone. The animals that had previously received the morphine/gefitinib combination retained a complete analgesic response to a subsequent dose of morphine (Fig. 2*A*,*B*). Since the half-life of gefitinib in rats is approximately 10 h (McKillop et al., 2004), this finding suggests that EGFR inhibition blocked the development of morphine tolerance. Of note, previous gefitinib administration did not alter the analgesic response to a probe dose of morphine on day 5 (Fig. 2*A*,*B*).

**Figure 3.**
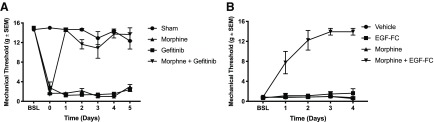
Inhibition of EGF signaling restores morphine analgesic effect against mechanical allodynia. ***A***, Following a two-week recovery after SNL or sham surgery, rats received daily intrathecal injections of either 1.5 μg morphine, 10 μg gefitinib, or MS + gefitinib. Mechanical sensitivity was tested after daily injections using the von Frey method. Day 0: baseline after two-week recovery; *N* = 6 rats per group, two-way ANOVA, interaction: *F*_(18,108)_ = 23.36, *p* < 0.0001; days: *F*_(6,108)_ = 75.96, *p* < 0.0001; treatment: *F*_(3,18)_ = 429.9, *p* < 0.001. ***B***, Following a two-week recovery after SNL, animals received daily intrathecal injections of either 1.5 μg morphine, 200 ng EGFR-Fc (EGF scavenging molecule), MS + EGFR-Fc, or vehicle. Mechanical sensitivity was tested daily. Day 0: postsurgical baseline; *N* = 6 rats per group, two-way ANOVA, interaction: *F*_(12,80)_ = 10.82, *p* < 0.0001; days: *F*_(4,80)_ = 17.92, *p* < 0.0001; treatment: *F*_(3,20)_ = 104.2, *p* < 0.0001.

These results suggested that EGFR activation was necessary for the development of morphine tolerance to occur. In order to determine whether EGFR activation itself was sufficient to induce tolerance, we determined whether repeated EGF administration would reduce the analgesic effect of a subsequent dose of morphine. EGF injections did not alter baseline tail flick responses. However, a dose of morphine administered after 4 d of EGF treatment did not elicit an analgesic response (vehicle: Captisol 10%, EGF: 63 ng, *N* = 6 rats per group, two-way ANOVA, interaction: *F*_(10,75)_ = 19.45, *p* < 0.0001; days: *F*_(5,75)_ = 20.43, *p* < 0.0001; treatment: *F*_(2,15)_ = 12.01, *p* < 0.0001; Fig. 3*C*), indicating that repeated EGF administration was sufficient to induce tolerance to a subsequent dose of morphine.

Opioids may have reduced effectiveness against neuropathic pain (Woolf and Mannion, 1999; Donica et al., 2014). Given that EGF induced tolerance to subsequent morphine doses, we wondered whether EGFR signaling could be responsible for this lack of efficacy. To test this hypothesis, we used the Chung SNL model (Chung et al., 2004). After a two-week recovery from the SNL procedure, animals received daily intrathecal injections of either vehicle, morphine, the EGFR inhibitor gefitinib or the combination of morphine and gefitinib. Mechanical allodynia was then assessed using the von Frey filament assay. In this paradigm, a substantial morphine dose did not relieve the mechanical sensitization (Fig. 3*A*). Gefitinib alone did not produce analgesia. However, combining gefitinib and morphine resulted in complete reversal of mechanical sensitization (vehicle: Captisol 10%, morphine: 1.5 μg, gefitinib: 10 μg, *N* = 6 rats per group, two-way ANOVA, interaction: *F*_(18,108)_ = 23.36, *p* < 0.0001; days: *F*_(6,108)_ = 75.96, *p* < 0.0001; treatment: *F*_(3,18)_ = 429.9, *p* < 0.001; Fig. 3*A*). Our findings suggest that EGFR signaling is both necessary and sufficient to cause opioid tolerance, and explain the morphine resistance often seen after nerve injury. EGFR activation after nerve injury could be due to either the release of EGF ligand from injured nerves or direct transactivation of the EGFR. To distinguish between these possibilities, we used the EGF scavenger construct EGFR-Fc, which consists of the extracellular domains of the EGFR fused to the Fc regions of immunoglobulin. Rats underwent SNL as above and then received daily intrathecal injections of vehicle, EGFR-Fc, morphine, or the combination of morphine and EGFR-Fc. EGFR-Fc alone had no analgesic effect. However, the combination of morphine and EGFR-Fc completely eliminated mechanical allodynia (vehicle: Captisol 10%, morphine: 1.5 μg, EGFR-Fc: 200 ng, *N* = 6 rats per group, two-way ANOVA, interaction: *F*_(12,80)_ = 10.82, *p* < 0.0001; days: *F*_(4,80)_ = 17.92, *p* < 0.0001; treatment: *F*_(3,20)_ = 104.2, *p* < 0.0001; Fig. 3*B*). This result suggested that EGFR ligands released from injured nerves induced tolerance to subsequent doses of morphine, in effect rendering animals pre-tolerant to opioids.

**Figure 4.**
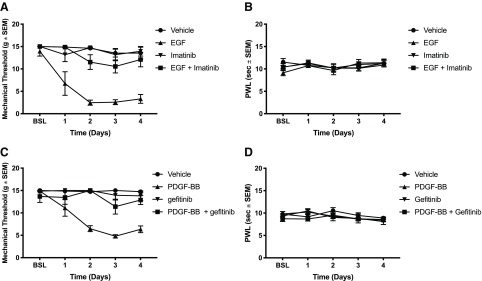
EGFR and PDGFR-β signaling interact in the production of mechanical allodynia. ***A***, Rats received daily intrathecal injections of either 63 ng EGF, 10 μg imatinib (PDGFR inhibitor), EGF + imatinib, or vehicle. Mechanical sensitivity was tested daily using the von Frey method; *N* = 6 rats per group, two-way ANOVA, interaction: *F*_(12,80)_ = 4.455, *p* < 0.0001; days: *F*_(4,80)_ = 13.21, *p* < 0.0001; treatment: *F*_(3,20)_ = 43.01, *p* < 0.0001. ***B***, Rats received daily intrathecal injections of either 63 ng EGF, 10 μg imatinib, EGF + imatinib, or vehicle. Thermal sensitivity was tested daily using the Hargreaves method; *N* = 6 rats per group, two-way ANOVA, interaction: *F*_(12,80)_ = 1.112, *p* = 0.3625, days: *F*_(4,80)_ = 2.745, *p* = 0.0340; treatments: *F*_(3,20)_ = 0.141, *p* = 0.9342. ***C***, Rats received daily intrathecal injections of either 250 ng PDGF-BB, 10 μg gefitinib (EGFR inhibitor), PDGF-BB + gefitinib, or vehicle. Mechanical sensitivity was tested daily; *N* = 6 rats per group, two-way ANOVA, interaction: *F*_(12,80)_ = 6.72, *p* < 0.0001; days: *F*_(4,80)_ = 11.12, *p* < 0.0001; treatment: *F*_(3,20)_ = 33.73, *p* < 0.0001. ***D***, Rats received daily intrathecal injections of either 250 ng PDGF-BB, 10 μg gefitinib, PDGF-BB+ gefitinib, or vehicle. Thermal sensitivity was tested daily; *N* = 4–5 rats per group, two-way ANOVA, interaction: *F*_(12,52)_ = 1.324, *p* = 0.2338, days: *F*_(4,52)_ = 4.353, *p* < 0.01; treatment: *F*_(3,13)_ = 0.7291.

Given the potential significance of these findings for understanding the mechanisms underlying neuropathic pain, we wanted to determine whether EGF administration would induce mechanical or thermal hypersensitivity. Others have shown that the related growth factor PDGF induced mechanical allodynia (Masuda et al., 2009). It had previously been demonstrated that PDGFR-β signaling also blocked opioid tolerance. Because of the similarities with EGF, we tested both EGF and PDGF in these paradigms. Animals received daily intrathecal injections of either EGF (Fig. 4*A*,*B*), PDGF-BB (Fig. 4*C*,*D*), or vehicle. Mechanical (Fig. 4*A*,*C*) and thermal (Fig. 4*B*,*D*) sensitivity were assessed daily after injection. We found that repeated injections of both EGF and PDGF-BB induced profound mechanical allodynia after 2 d [vehicle: Captisol 10%, EGF: 63 ng, imatinib: 10 μg, *N* = 6 rats per group, two-way ANOVA, interaction: *F*_(12,80)_ = 4.455, *p* < 0.0001; days: *F*_(4,80)_ = 13.21, *p* < 0.0001; treatment: *F*_(3,20)_ = 43.01, *p* < 0.0001 (Fig. 4*A*) and vehicle: Captisol 10%, PDGF-BB: 250 ng, gefitinib: 10 μg, *N* = 6 rats per group, two-way ANOVA, interaction: *F*_(12,80)_ = 6.72, *p* < 0.0001; days: *F*_(4,80)_ = 11.12, *p*< 0.0001; treatment: *F*_(3,20)_ = 33.73, *p* < 0.0001 (Fig. 4*C*)]. However, thermal hypersensitivity was not observed after multiple EGF or PDGF-BB injections [vehicle: Captisol 10%, EGF: 63 ng, imatinib: 10 μg, *N* = 6 rats per group, two-way ANOVA, interaction: *F*_(12,80)_ = 1.112, *p* = 0.3625, days: *F*_(4,80)_ = 2.745, *p* = 0.0340; treatments: *F*_(3,20)_ = 0.141, *p* = 0.9342 (Fig. 4*B*) and vehicle: Captisol 10%, PDGF-BB: 250 ng, gefitinib: 10 μg, *N* = 4–5 rats per group, two-way ANOVA, interaction: *F*_(12,52)_ = 1.324, *p* = 0.2338, days: *F*_(4,52)_ = 4.353, *p* < 0.01; treatment: *F*_(3,13)_ = 0.7291 (Fig. 4*D*)].

In previous studies, the PDGFR-β and the EGFR have been shown to interact (Habib et al., 1998; Saito et al., 2001). Given this fact and our observation that EGF and PDGF-BB both caused mechanical allodynia, we investigated whether EGF and PDGF-BB could interact to produce mechanical allodynia. Animals were injected daily with either EGF, the PDGFR-β inhibitor imatinib, EGF + imatinib, or vehicle. Remarkably, the PDGFR-β inhibitor imatinib prevented EGF-induced mechanical allodynia (Fig. 4*A*). In another experiment, animals were injected with either PDGF-BB, the EGFR inhibitor gefitinib, PDGF-BB + gefitinib, or vehicle. Similarly, the EGFR inhibitor gefitinib blocked the development of PDGF-BB-induced mechanical allodynia (Fig. 4*C*). Thermal sensitization was not induced by these treatments (Fig. 4*D*). These results indicate that EGFR and PDGFR-β signaling are intimately linked in the production of mechanical hypersensitivity.

## Discussion

In this study, we demonstrated that the EGFR co-localized with multiple classes of neurons in the DRG and selectively co-localized with CGRP, IB4, and some NF200 expressing primary afferent fibers in the SG, as well as in scattered microglial cells. Previous studies have yielded similar results (Werner et al., 1988; Huerta et al., 1996; Martin et al., 2017). The MOR is known to be expressed in peptidergic primary afferent fibers in the SG and in DRG peptidergic neurons (Scherrer et al., 2009). The EGFR has also been shown to be activated by the MOR (Belcheva et al., 2001, 2003). EGFR signaling has been shown to activate the microglial inflammatory response (Qu et al., 2012), and microglia have been shown to play a role in morphine tolerance (Hutchinson et al., 2011; Burma et al., 2017). Taken together, these results provide an anatomic substrate for the behavioral findings we observed.

We showed that EGFR signaling is both necessary and sufficient to cause opioid tolerance. When EGFR signaling was inhibited, morphine tolerance was not observed. Conversely, after chronic EGF administration complete tolerance to a subsequent dose of morphine was seen although the animals had never received morphine before. This is similar to previous observations of the effects of PDGFR-β modulation on tolerance (Wang et al., 2012). However, there is a key difference between these two findings. After daily doses of morphine and the PDGFR inhibitor imatinib, which cause sustained analgesia, a dose of morphine alone was not analgesic. In the present study, when morphine alone was administered 1 d after repeated doses of morphine and gefitinib, the animals had a full analgesic response. The half-life of gefitinib in rodents is approximately 4–6 h (McKillop et al., 2004; Maher et al., 2016) and the half-life of imatinib is approximately 12 h (Bende et al., 2010), indicating that neither drug is likely exerting sustained pharmacologic effects. Taken together, these observations suggest that EGFR inhibition by gefitinib blocks the mechanistic processes causing the development of tolerance, while PDGFR-β inhibition by imatinib does not block the development of tolerance, but rather temporarily reverses or bypasses these mechanisms, blocking the behavioral expression of tolerance. Previous work with the PDGFR-β (Wang et al., 2012) also demonstrated that morphine tolerance can be selectively targeted without affecting its analgesic properties. Recent studies also further support the possibility of selectively targeting opioid side effects (Burma et al., 2017; Corder et al., 2017).

We also discovered that while EGFR inhibition was not analgesic against neuropathic pain, combining EGFR inhibition with a previously ineffective dose of morphine resulted in a robust analgesic effect. This restoration of the analgesic effect of morphine was also observed with the EGFR-Fc construct, an EGF scavenger. This finding suggests that the injured nerves may release EGF, rendering the animals less responsive or pre-tolerant to the analgesic effect of morphine. This may explain the observation that clinical neuropathic pain is often very difficult to treat with opioids (Woolf and Mannion, 1999). In contrast to our findings, a recent study by Martin et al suggested that gefitinib was analgesic in the spared nerve injury (SNI) model of neuropathic pain (Martin et al., 2017). In that study, gefitinib alone partially alleviated neuropathic allodynia, but required gefitinib doses 6–60 times higher than those used in our study. This discrepancy could potentially be due to an off-target action of gefitinib or reflect differences between the SNI and the SNL neuropathic pain model we used (Colleoni and Sacerdote, 2010). We also found that chronic, but not acute, injections of EGF caused the development of a robust mechanical allodynia. Consistent with our results, Martin and collaborators reported that acute EGF injection did not increase mechanical sensitivity (Martin et al., 2017). They did not investigate chronic EGF injection. We found that it takes several days for EGF to induce mechanical hypersensitivity in naive rats. Consistent with previous studies, chronic but not acute injections of PDGF-BB also caused mechanical hypersensitivity (Masuda et al., 2009).

**Figure 5.**
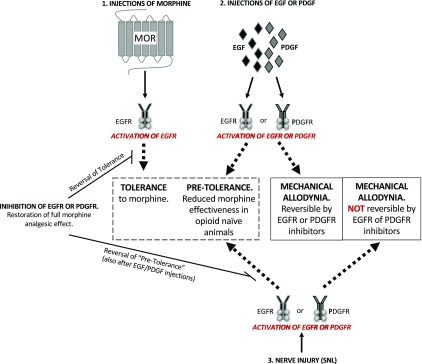
Graphic summary of findings. Pathway 1 shows that morphine administration causes tolerance by activation of the EGFR. We have shown that morphine administration causes tolerance by activating the EGFR. EGFR (or PDGFR) inhibition eliminates or reverses analgesic tolerance, restoring or maintaining the full analgesic effect of morphine. Pathway 2 details the effects of EGF administration on tolerance and mechanical sensitivity. Giving EGF (or PDGF) to animals chronically induces mechanical allodynia and reduces the analgesic effectiveness of a subsequent dose of morphine, in effect causing a pre-tolerant state in the absence of prior opioid exposure. Both of these phenomena can be blocked or reversed by EGFR or PDGFR inhibition. Pathway 3 shows that nerve injury induces release of EGF and PDGF, which subsequently activates the EGFR and PDGFR and induces mechanical allodynia. However, EGFR or PDGFR inhibition does not reverse the allodynia caused by nerve injury. This is likely due to the concomitant activation of a variety of other mediators of allodynia by nerve injury. Nerve injury also induces a pre-tolerant state that can be eliminated or reversed by EGFR or PDGFR inhibition, suggesting that pre-tolerance is selectively mediated by EGFR and/or PDGFR signaling. In sum, we can conclude that EGFR and PDGFR signaling mediate morphine analgesic tolerance and can induce a pre-tolerant state in opioid-naive animals. While EGFR and PDGFR signaling can induce mechanical allodynia, allodynia induced by nerve injury appears to involve a more complex set of mediators. However, EGFR or PDGFR inhibition still selectively reverse the decrease in morphine analgesia induced by nerve injury (pre-tolerance), suggesting that using these inhibitors clinically could permit the sustained treatment of nerve injury pain using far lower doses of opioids for extended periods of time.

Strikingly, although imatinib and gefitinib could prevent mechanical hypersensitivity caused by peptide injections, they could not relieve SNL-induced mechanical allodynia when injected without morphine (Fig. 3*A*; Donica et al., 2014). Scavenging released EGF in the SNL model also did not reduce allodynia (Fig. 3*B*). Neuropathic pain-induced mechanical allodynia is known to involve a plethora of effectors including cytokines, chemokines, prostaglandins, histamine and other growth factors (Moalem and Tracey, 2006; Chen et al., 2018). These complex neurochemical responses likely induce nociceptive responses not duplicated by growth factor injection alone. Our results suggest that while growth factors could play a role in the initiation of nerve injury induced allodynia, as the condition progresses, multiple additional effectors sustain the pain. However, our findings also indicate that the allodynia induced by neuropathic pain could be readily treated by morphine and potentially other opioids if the opioid tolerant state induced by growth factor release (pre-tolerance) is reversed by concomitant administration of imatinib or gefitinib. Figure 5 presents a schematic summary of our findings.

Interestingly, neither EGF nor PDGF-BB caused thermal hypersensitivity, showing that their involvement in pain signaling is modality specific. This result also indicates that the diminution of opioid analgesic effects by chronic administration of EGF (Fig. 2*C*) or PDGF was not likely due to the development of opioid-induced hyperalgesia (Angst and Clark, 2006), as chronic administration of these growth factors never induced thermal hyperalgesia. Behavioral responses to noxious mechanical and thermal stimulation have been thought to be conveyed by distinct subsets of primary afferent neurons that are distinguished by specific modality-related cellular markers (Cavanaugh et al., 2009; Scherrer et al., 2009). We have shown that EGFR is expressed by the majority, if not all, DRG sensory neurons in rat, while others have demonstrated similar expression patterns in mouse (Koprivica et al., 2005; Maklad et al., 2009; Martin et al., 2017), and human (Werner et al., 1988; Huerta et al., 1996). PDGFR-β has also been shown to be widely expressed in DRG neurons (Sasahara et al., 1991; Eccleston et al., 1993). Therefore, the modality specific sensitization induced by growth factor signals cannot be simply explained by their pattern of expression in DRG primary sensory neurons. Alternatively, the population-coding theory hypothesizes that different modalities are processed by distinct molecularly defined cell types in the spinal cord (Ma, 2012). In this theory, molecularly defined DRG sensory neurons connect spinally with discrete neural circuits that, when activated, generate a specific sensation (Peirs and Seal, 2016; Duan et al., 2018; Koch et al., 2018). Under physiological conditions, the classic gate control theory describes that light touch, generated by low-threshold mechanosensory primary afferent A-fibers, inhibits pain through the activation of a feed-forward inhibitory circuit in the dorsal horn of the spinal cord (Melzack and Wall, 1965). However, in the event of injury, this feed forward circuit is thought to be impaired. Light touch then engages a polysynaptic spinal nociceptive network that activates the pain projection neurons in the dorsal horn of the spinal cord, which causes mechanical allodynia (for review, see Peirs and Seal, 2016). Whether growth factors mediate mechanical allodynia by activating this circuit remains to be determined.

Our work has shown that the EGFR is a core mediator of analgesic tolerance. Other RTKs, namely, PDGFR-β (Wang et al., 2012), fibroblast growth factor receptor (FGFR; Fujita-Hamabe et al., 2011), and Ephrin receptor (EphB; Liu et al., 2011), have been shown to modulate morphine analgesic tolerance. These RTKs are closely phylogenetically related to the EGFR (Brunet et al., 2016). Thus, RTK signaling could play a prominent role in mediating opioid tolerance. In support of this idea, RTKs have been shown to activate G-protein-coupled receptors (GPCRs; Delcourt et al., 2007; Di Liberto et al., 2019). The MOR is a GPCR, and it has been shown that EGFR-mediated phosphorylation can reduce MOR signaling (Clayton et al., 2010). Future studies to further elucidate the mechanisms by which RTKs modulate opioid signaling and the generalizability of this phenomenon could have important clinical implications.

In conclusion, we have shown that EGFR activation is not only necessary and sufficient to cause morphine tolerance, but also induces mechanical allodynia and opioid resistance in a nerve injury model. The fact that EGF induced morphine tolerance, opioid resistance as well as a robust mechanical allodynia establishes a key mechanistic link between pain and opioid tolerance. Our work suggests that EGFR inhibition could not only improve the effectiveness of opioids against neuropathic pain but could also limit the dose escalation leading to increased incidence of undesirable and potentially life-threatening side effects of opioid use.

## Synthesis

Reviewing Editor: Karen Szumlinski, University of California at Santa Barbara Decisions are customarily a result of the Reviewing Editor and the peer reviewers coming together and discussing their recommendations until a consensus is reached. When revisions are invited, a fact-based synthesis statement explaining their decision and outlining what is needed to prepare a revision will be listed below. The following reviewer(s) agreed to reveal their identity: Theodore Price.

Upon consultation with the two expert reviewers of the original report, I am pleased to inform you that we now find your report acceptable for eNeuro. We appreciate your efforts addressing the reviewers' comments.
